# P-1372. Text Message Contact Tracing Intervention for Tuberculosis Screening in South Africa: A Cluster Randomized Controlled Trial

**DOI:** 10.1093/ofid/ofaf695.1559

**Published:** 2026-01-11

**Authors:** Colin McLeish, Leisha Genade, Floris J C Swanepoel, Minja Milovanovic, Bareng Aletta Sanny Nonyane, Khuthadzo Hlongwane, Limakatso Lebina, Neil Martinson

**Affiliations:** Johns Hopkins University School of Medicine, Baltimore, MD; University of the Witwatersrand, Johannesburg, Gauteng, South Africa; University of the Witwatersrand, Johannesburg, Gauteng, South Africa; African Potential Group, Johannesburg, Gauteng, South Africa; Johns Hopkins Bloomberg School of Public Health, Baltimore, Maryland; University of the Witwatersrand, Johannesburg, Gauteng, South Africa; Africa Health Research Institute, Mtubatuba, KwaZulu-Natal, South Africa; University of the Witwatersrand, Johannesburg, Gauteng, South Africa

## Abstract

**Background:**

Tuberculosis (TB) contact tracing identifies and refers individuals exposed to TB for screening. Notification methods include phone calls, clinic referral cards, and text messages. We evaluated whether offering a text message notification option could increase the proportion of contacts presenting for TB screening in South Africa.

**Methods:**

We performed a cluster-randomized trial at 29 primary care clinics in 3 provinces of South Africa from October 2019 to March 2020, nested in the Targeted Universal Testing for TB (TUTT) trial. Clinics were randomized in a 2:1 ratio to: (i) offer index patients two options of text message notifications—sent from their own mobile device or by clinic staff—as an additional or alternative method to clinic referral cards (intervention), or (ii) provide clinic referral cards only (control) to notify contacts. The primary outcome was the cluster-level proportion of contacts presenting for TB screening.

**Results:**

A total of 620 index patients diagnosed with TB (56.9% male; median age 36) identified 2,058 close contacts (median 3.0 per index; IQR 1.5-5.0).

Of the 505 index patients in the intervention arm, 302 (59.8%) exclusively used clinic referral cards to notify contacts; 97 (19.2%) had clinic staff send the contact message; 70 (13.8%) used text messages from clinic staff and clinic referral cards; 3 (0.6%) sent text messages from their personal devices. Preferred method of notification did not vary significantly by age group (P=0.264, Chi square).

In the 18 intervention group clinics, 10.2% (168/1653) of contacts presented for screening across all methods of referral, compared to 9.6% (39/405) of contacts in the 11 control group clinics (P=0.749, Z test).

In the intervention arm, 1275 clinic referral cards and 378 text messages were distributed, of which 12.2% (155/1275) and 3.4% (13/378) were, respectively, reported to have been received by contacts presenting for screening (P< 0.001, Z test).

**Conclusion:**

Augmenting clinic referral cards with a text message option did not affect the proportion of contacts presenting for TB screening in a high-prevalence setting. Clinic referral cards, which were preferred by index patients with TB and attracted more contacts than text messages, should be implemented as an inexpensive method to bring contacts for assessment.

**Disclosures:**

All Authors: No reported disclosures

